# Clinical Implications of Isocitrate Dehydrogenase Mutations and Targeted Treatment of Acute Myeloid Leukemia with Mutant Isocitrate Dehydrogenase Inhibitors—Recent Advances, Challenges and Future Prospects

**DOI:** 10.3390/ijms25147916

**Published:** 2024-07-19

**Authors:** Adrian Kowalczyk, Julia Zarychta, Monika Lejman, Eryk Latoch, Joanna Zawitkowska

**Affiliations:** 1 Student Scientific Society of Department of Pediatric Hematology, Oncology and Transplantology, Medical University of Lublin, 20-093 Lublin, Poland; adriankowalczyk31@gmail.com (A.K.); julia.zarychta99@gmail.com (J.Z.); 2 Independent Laboratory of Genetic Diagnostics, Medical University of Lublin, 20-093 Lublin, Poland; monika.lejman@umlub.pl; 3 Department of Pediatric Oncology and Hematology, Medical University of Bialystok, 15-274 Bialystok, Poland; eryk.latoch@umb.edu.pl; 4 Department of Pediatric Hematology, Oncology and Transplantology, Medical University of Lublin, 20-093 Lublin, Poland

**Keywords:** acute myeloid leukemia, isocitrate dehydrogenase inhibitors, ivosidenib, enasidenib, targeted therapy

## Abstract

Despite the better understanding of the molecular mechanisms contributing to the pathogenesis of acute myeloid leukemia (AML) and improved patient survival in recent years, AML therapy still remains a clinical challenge. For this reason, it is important to search for new therapies that will enable the achievement of remission. Recently, the Food and Drug Administration approved three mutant IDH (mIDH) inhibitors for the treatment of AML. However, the use of mIDH inhibitors in monotherapy usually leads to the development of resistance and the subsequent recurrence of the cancer, despite the initial effectiveness of the therapy. A complete understanding of the mechanisms by which *IDH* mutations influence the development of leukemia, as well as the processes that enable resistance to mIDH inhibitors, may significantly improve the efficacy of this therapy through the use of an appropriate synergistic approach. The aim of this literature review is to present the role of *IDH1*/*IDH2* mutations in the pathogenesis of AML and the results of clinical trials using mIDH1/IDH2 inhibitors in AML and to discuss the challenges related to the use of mIDH1/IDH2 inhibitors in practice and future prospects related to the potential methods of overcoming resistance to these agents.

## 1. Introduction

Acute myeloid leukemia (AML) is the second most common hematologic cancer in children and the most common type of acute leukemia in the adult population. The average age of onset of AML in patients is approximately 68–71 [1]. Despite the better understanding of the molecular mechanisms contributing to the pathogenesis of AML and the improved patient survival in recent years (the 5-year relative survival rate in 1996 was 17.4%, while that in 2016 was 31.6%), AML therapy still remains a clinical challenge [2]. The standard treatment is intensive induction chemotherapy to achieve remission, followed by consolidation chemotherapy to maintain remission and achieve recovery. In the case of patients belonging to the increased risk group, after achieving remission, an allogeneic hematopoietic stem cell transplant (allo-HSCT) is performed. Due to comorbidities in the elderly population, not all patients qualify for intensive treatment, and, even if intensive treatment is used, it is estimated that approximately 10–40% of young patients and 40–60% of patients over 60 years of age will not respond to the treatment therapy [1,3,4,5]. Additionally, despite a good response to induction therapy, it is estimated that over 50% of patients after the consolidation phase will experience a relapse of the disease within 6–9 months [6]. AML recurrence or resistance to treatment is associated with a poor prognosis (median overall survival is estimated at approximately 6 months after the initiation of rescue treatment) [5,7]. One of the possible therapy options for such patients is allo-HSCT; however, it should be performed in patients in remission, because the likelihood of the therapy being successful is the highest at this time [7]. For this reason, it is important to search for new targeted therapies that will enable the achievement of remission in both geriatric patients who do not qualify for intensive treatment because of their comorbidities and patients with relapsed or refractory (R/R) AML. In these cases, the use of a personalized approach will enable the achievement of remission, which is necessary to perform allo-HSCT, a rescue procedure that improves survival in this group of patients [4,7].

Due to the dysregulation of the molecular pathways involved in the pathogenesis of AML, the use of mitogen-activated protein extracellular kinase 1/2 (MEK 1/2) and mammalian target of rapamycin (mTOR) inhibitors in AML therapy has been evaluated, but clinical trials have not achieved satisfactory clinical effectiveness with the aforementioned agents [8,9]. Another analyzed molecular target, the inhibition of which could improve the results of AML therapy, consists of mutations in the genes encoding isocitrate dehydrogenase isoform 1 (IDH1) and isocitrate dehydrogenase isoform 2 (IDH2), occurring at a frequency of 8–17% and 12–19% of AML cases, respectively [10,11,12,13,14]. The frequency of IDH mutations increases with the age of the patients. For example, *IDH1* mutations in the population of young AML patients are found in approximately 6–10% of cases, while, in geriatric patients, the frequency of *IDH1* mutations is estimated at approximately 17% [12]. Although the occurrence of *IDH* mutations alone seems to be insufficient to initiate the development of leukemia, the mere presence of *IDH* mutations leads to a block in the differentiation of hematopoietic stem cells and promotes the occurrence of further mutations that may initiate the cancer process [12,15]. In addition to the presence of *IDH* mutations in leukemic blasts, these mutations have also been identified in the population of pre-leukemic hematopoietic stem cells, which, if not eliminated during therapy, constitute a reservoir for the disease and may be a potential cause of relapse [16].

The aim of this literature review is to present the role of *IDH1* and *IDH2* mutations in the pathogenesis of AML and the results of clinical trials using mutant IDH1 (mIDH1) and mutant IDH2 (mIDH2) inhibitors in newly diagnosed and R/R AML, and to discuss the challenges related to the use of mIDH1 and mIDH2 inhibitors in practice (the mechanisms behind the development of resistance to treatment and the side effects of the drugs) and future prospects related to the potential methods of overcoming resistance to treatment with mIDH1 and mIDH2 inhibitors.

## 2. The Role of IDH Mutations in the Pathogenesis of AML

**Figure 1:**
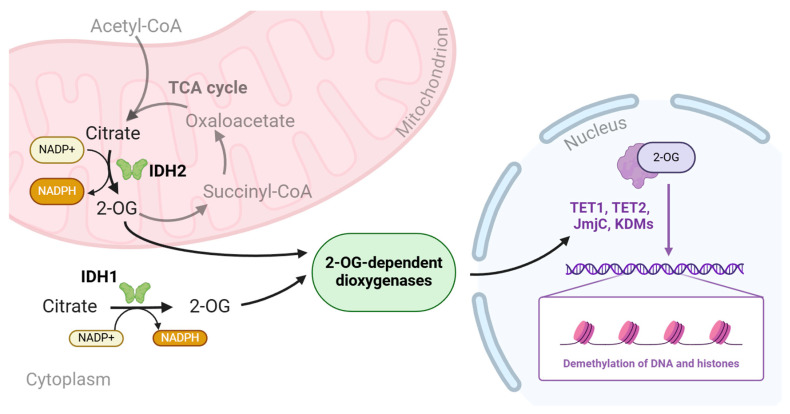
Wild-type IDH1 and IDH2 catalyze the reaction of converting citrate to 2-oxoglutarate (2-OG). Physiologically, 2-OG, after penetrating the cell nucleus, is involved in the activation of dioxygenases, which play a role in the process of deoxyribonucleic acid (DNA) and histone demethylation. IDH1—isocitrate dehydrogenase 1, IDH2—isocitrate dehydrogenase 2, 2-OG—2-oxoglutarate, NADPH—nicotinamide adenine dinucleotide phosphate, TCA—tricarboxylic acid, CoA—coenzymeA, TET1/TET2—ten-eleven translocation 1/2, JmjC—Jumonji-C family, KDMs—histone lysine demethylases, DNA—deoxyribonucleic acid. Image created with biorender.com (accessed on 19 June 2024).

Physiologically, IDH1 and IDH2 isoenzymes contribute to the regulation of cellular metabolism in various intracellular compartments (IDH1 localizes in the cytoplasm and peroxisomes, while IDH2 exhibits catalytic activity in mitochondria)—see Figure 1.

In addition to IDH1 and IDH2, there is another isoform of this enzyme—IDH3. Unlike isoforms 1 and 2, discussed more broadly here, IDH3 is NAD-dependent. Mutations in the gene encoding this isoform have been detected in various types of cancer, but so far it has not been proven that they affect carcinogenesis. This is probably due to the nature of the mutations, which, unlike IDH1 and IDH2 mutations, are primarily inactivating mutations and, therefore, do not result in the cancer cell acquiring new functions [17].

The *IDH1* and *IDH2* mutations found in AML belong to the so-called gain of function (GOF) mutations [18]. The most frequently described changes concern the substitution of a single arginine residue (R) in the active site of the enzyme: *IDH1^R132^*, *IDH2^R140^* and *IDH2^R172^* [19,20,21]. The *IDH2^R172^* mutation often co-occurs with the *DNMT3A* mutation, while the *IDH2^R140^* mutation often co-occurs with the *NPM1*, *SRSF2* and *DNMT3A* mutations [21].

Thanks to the GOF mutation, IDH1 and IDH2 acquire the neomorphic ability to convert 2-oxoglutarate (2-OG) to the oncometabolite 2-hydroxyglutarate (2-HG) [10,22]. The abnormally increased pool of intracellular 2-HG is caused by two mechanisms: (i) the preferential conversion of 2-OG to 2-HG by mutant IDH1/IDH2 (instead of catalyzing the reaction of converting 2-OG to D-isocitrate) and (ii) the activity of intracellular D-2-hydroxyglutarate dehydrogenase, which catalyzes the reaction of converting 2-HG to 2-OG, inadequately with regard to the increased 2-HG concentration [23,24]. The increased production of 2-HG and its insufficient removal, leading to the accumulation of oncometabolites in the cell, promotes oncogenesis in many ways [24].

### 2.1. Epigenetic Modifications

Physiologically, 2-OG, after penetrating the cell nucleus, is involved in the activation of dioxygenases, which play a role in the process of deoxyribonucleic acid (DNA) and histone demethylation. Here, 2-HG competes with 2-OG for the binding site with DNA demethylases from the ten-eleven translocation (TET) family and Jumonji family histone lysine demethylases (JmjC KDMs) [23]. The increased concentration of 2-HG in cancer cells promotes the binding of TET or JmjC KDMs to 2-HG. It should be noted, however, that 2-HG does not have the ability to activate these enzymes. Instead, it leads to less active TET or JmjC KDM function, which results in the hypermethylation of DNA and histones. This, in turn, disturbs gene expression and thus blocks the process of the proper differentiation of hematopoietic stem and progenitor cells [10,12,19,21,25]. Studies have shown that, out of the three TET isoforms, the functions of the TET1 and TET2 isoforms are more affected by the 2-HG increase [26,27]. Changes in the chromatin structure, contributing to the blocking of the normal differentiation of hematopoietic stem cells, may be associated with the higher percentage of blasts found in the peripheral blood and bone marrow in patients with the *IDH* mutation [21,23].

The mTOR pathway is also involved in promoting tumorigenesis, the dysregulation of which in *IDH*-mutated cells is caused by the inhibition of one of the JMJC KDMs, lysine-specific demethylase 4A (KDM4A), by 2-HG. Reduced KDM4A activity contributes to the reduced expression and stability of the negative regulator of the mammalian target of rapamycin complex 1/2 (mTORC1/2) pathway, which ultimately leads to the increased activity of the mTOR pathway (independently of the phosphatidylinositol-3 kinase (PI3K)/ protein kinase B (AKT)/tuberous sclerosis complex 1–2 (TSC1–2) pathway) in cells affected by the *IDH* mutation [6].

Epigenetic modifications also cause changes in the expression of oncogenes and tumor suppressor genes in cells affected by *IDH* mutations. For example, Steinhäuser et al. showed that the *IDH1^R132H^* mutation promotes the expression of the oncogene *PDGFRA* [12]. Physiologically, gene expression is coordinated within topologically associated domains (TADs). Individual TADs separate 11-zinc finger protein (CTCF) proteins from each other. The hypermethylation of the CpG site in the CTCF region upstream of the *PDGFRA* gene locus, caused by the increased concentration of 2-HG, contributes to a decrease in the binding strength of CTCF to DNA, thereby disrupting the TAD structure, which ultimately leads to the incorrect interaction of the enhancer with the *PDGFRA* promoter, leading to the upregulation of the oncogene in AML cells with an *IDH1^R132H^* mutation [12,28]. Increased chromatin methylation also leads to the decreased expression of the tumor suppressor gene *TP53* [29]. The *IDH* mutation also contributes to the promotion of *DOT1L* overexpression, which ultimately results in the increased expression of *HOXA9* and *MEIS1* and further promotes malignant transformation through the subsequent activation of the expression of genes supporting proliferation and inhibiting differentiation [30,31].

### 2.2. Accumulation of Mutations

**Figure 2:**
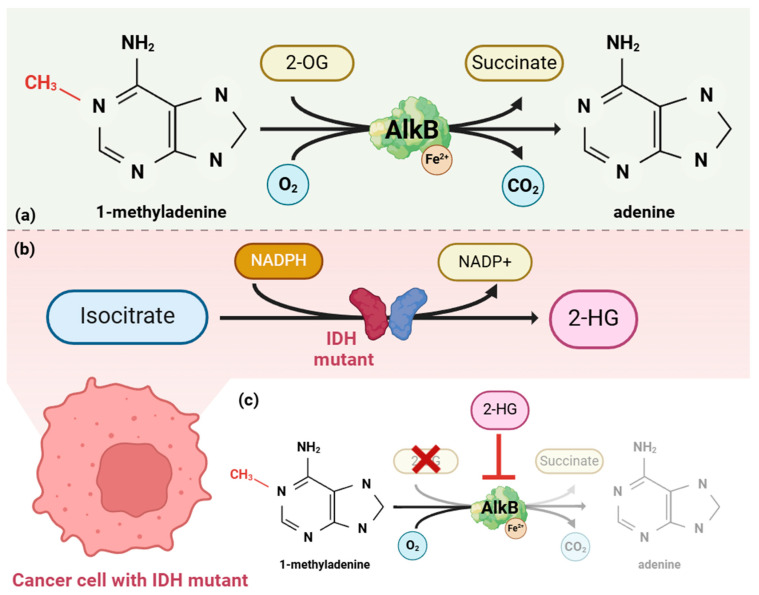
(**a**) AlkB plays a role in the repair of alkylated DNA by converting mutagenic 1-methyladenine/3-methylcytosine into normal A/C bases. One of the substrates necessary to carry out the reaction is 2-OG. (**b**) In cancer cells with an *IDH* mutation, 2-HG is produced instead of 2-OG in the reaction catalyzed by the mutant enzymes. (**c**) Due to 2-OG deficiency and an increased intracellular 2-HG concentration, which is an AlkB inhibitor, there is the accumulation of methylated damage in the DNA, which may ultimately lead to the formation of further mutations in the cancer cell. 2-OG—2-oxoglutarate, NADPH—nicotinamide adenine dinucleotide phosphate, IDH—isocitrate dehydrogenase, 2-HG—2-hydroxyglutarate, DNA—deoxyribonucleic acid. Image created with biorender.com (accessed on 19 June 2024).

In addition to inhibiting dioxygenases involved in the process of DNA and histone demethylation, 2-HG also inhibits enzymes from the AlkB homolog (ALKBH) family [23]. Physiologically, ALKBHs play a role in the repair of alkylated DNA by converting mutagenic 1-methyladenine/3-methylcytosine into normal A/C bases. One of the substrates required for this transformation is 2-OG—see Figure 2 [32,33].

An insufficient amount of intracellular 2-OG and an excess of 2-HG, caused by the abnormal activity of mutant IDH, leads to the accumulation of methylated damage in DNA, which may ultimately lead to the formation of further mutations that are crucial for the pathogenesis of leukemia [23]. Additionally, abnormal histone hypermethylation and the inhibition of 2-OG-dependent dioxygenases (KDM4A and lysine-specific demethylase 4B—KDM4B) limit the efficiency of the repair of double-stranded DNA breaks in the process of homologous recombination [23,30].

### 2.3. Increased Susceptibility to Oxidative Stress

Oxidative decarboxylation, during which D-isocitrate is converted to 2-OG, is one of the sources enabling the regeneration of the cellular nicotinamide adenine dinucleotide phosphate (NADPH) pool. The reducing potential of NADPH is used, among other aspects, to regenerate glutathione (GSH), which, by participating in maintaining the intracellular oxidation–reduction balance, counteracts the adverse effects of oxidative stress [23,34]. As a result of mutations, IDHs acquire the ability to convert 2-OG into 2-HG, and, in order to carry out this reaction, it is necessary to consume the intracellular NADPH pool, which contributes to the greater sensitivity of the cell to oxidative stress [23]. A decrease in intracellular NADPH and an increase in NADP promote an increased amount of reactive oxygen species in the cell. This process is additionally intensified by the slowdown of mitochondrial metabolism (resulting from the increased concentration of 2-HG and the activation of the mTOR pathway) and the associated dysregulation of the electron transport chain, resulting in electron leakage [6].

The increased susceptibility to oxidative stress of cells with the *IDH* mutation is also caused by changes in amino acid metabolism. Physiologically, glutamate is used to produce the previously mentioned GSH, which ensures an oxidation–reduction balance. One of the sources of glutamate in the cell is the transamination reaction of branched-chain amino acids to branched-chain α-keto acids. One of the substrates of this reaction is 2-OG, which is converted into glutamate during the reaction. A decreased intracellular 2-OG concentration disrupts the metabolism of branched-chain amino acids, thereby reducing the pool of intracellular glutamate used for the production of GSH [23]. Another source of glutamate is the reaction catalyzed by mitochondrial glutaminase (GLS), during which glutamine is converted to glutamate. Cancer cells often overexpress GLS and develop a dependence on glutamine, the transformation of which can be a source of both GSH and 2-OG [30]. A possible compensatory mechanism for the maintenance of the intracellular GSH pool is the increased activity of nuclear factor erythroid 2-related factor 2 (Nrf2), which in turn promotes the expression of the gene encoding the enzyme cysteine–glutamine ligase, involved in the synthesis of GSH [30].

### 2.4. Alteration of Cellular Metabolism and Reduction of Hypoxia-Induced Factor 1α Activity (HIF-1α)

The *IDH1*/*IDH2* mutation also causes changes in cellular metabolism. The reduced concentration of 2-OG in the cell, resulting from its transformation by mIDH into 2-HG, results in the reduced effectiveness of the Krebs cycle (2-OG is one of the substrates of the Krebs cycle) [35]. Additionally, 2-HG has an inhibitory effect on the enzymes catalyzing the Krebs cycle: fumarate hydratase and succinate dehydrogenase [36]. In turn, reducing the activity of 2-OG-dependent dioxygenases involved in lipid metabolism reduces the synthesis of phospholipids in cells affected by the mutation [23]. The disorder of lipid metabolism is further aggravated by the intracellular deficit of NADPH and isocitrate, which can be used for de novo lipid synthesis [37]. On the other hand, the IDH mutation contributes to the promotion of oxidative phosphorylation (OxPHOS) and fatty acid β-oxidation (FAO) [6]. The increased oxidative metabolism may be due to 2-HG-induced epigenetic dysregulation, resulting in increased CCAAT/enhancer-binding protein alpha (CEBPα) methylation [38].

Another dioxygenase, the activity of which is dependent on 2-OG, is prolyl hydroxylase (PHD) [30]. Physiologically, PHD regulates hypoxia-inducible factor 1-alpha (Hif-1α) stability. Under aerobic conditions, PHD promotes the degradation of Hif-1α, while hypoxic conditions inhibit PHD activity [39,40,41]. By binding to PHD, 2-HG reduces its activity in aerobic conditions, which leads to the upregulation of Hif-1α and its dependent gene expression; this is involved in angiogenesis, glucose metabolism, cell proliferation and survival, among others [30,39,40,41]. Although this process has been described mainly in *IDH*-mutant glioma cell models, it is possible that increasing Hif-1α’s activity promotes the pathogenesis of AML, but this requires further research [2].

### 2.5. Reduction of Apoptosis of Leukemic Cells

Moreover, 2-HG, by exerting an inhibitory effect on cytochrome C oxidases, contributes to increasing the expression of the anti-apoptotic protein B-cell leukemia/lymphoma 2 protein (BCL-2) in cancer cells, which ultimately leads to a reduction in apoptosis [35]. Increasing the expression of BCL-2 may be a compensatory mechanism of cancer cells, thanks to which apoptosis does not occur after reducing the activity of cytochrome C oxidase. However, a better understanding of this relationship requires further research [42].

### 2.6. Promoting Tumor Cell Proliferation and the Immunosuppressive Phenotype of the Tumor Microenvironment (TME)

The role of 2-HG in shaping the TME phenotype in AML is not clear. Extracellular 2-HG (secreted paracrine by tumor cells) may lead to the activation of nuclear factor kappa B (NF-κB)-dependent transcription factors in bone marrow stromal cells, thereby contributing to the production of cytokines and adhesion molecules promoting blast proliferation and survival [23]. For example, interleukin 6, secreted by bone marrow stromal cells, may support the proliferation of cancer cells, while the presence of adhesion molecules determining cell–cell interactions may promote the chemoresistance of blasts [6].

It has been reported that 2-HG may, by influencing HIF signaling in the Th17 lymphocytes present in the TME, disturb their differentiation [6]. An increased concentration of extracellular 2-HG may also affect the effector functions of CD8^+^ T lymphocytes infiltrating cancer cells by weakening their proliferation and reducing the production of interferon γ via the direct inhibition of the enzymatic activity of lactate dehydrogenase (LDH) of T lymphocytes. This leads to disturbed glucose metabolism in these cells, related to the reduced efficiency in converting pyruvate into lactate in the process of anaerobic glucose metabolism [6,43]. However, LDH also catalyzes the reverse reaction, during which lactate is converted to pyruvate. Decreased LDH activity, resulting from an abnormal 2-HG concentration in the cell, may also potentially contribute to an increased intracellular lactate concentration, which is another key oncometabolite. Lactate, by acidifying the TME, may additionally impair the function of immune cells [44]. However, determining the effect of 2-HG on the concentration of lactate in the TMEs of tumors with IDH mutations requires further research. Due to the effect of 2-HG on the TME in *IDH*-mutated gliomas, it is possible that 2-HG also contributes to the immunosuppressive phenotype of the TME in AML; however, the confirmation of this hypothesis requires further research covering, among other aspects, the mechanism of 2-HG transport to the TME [23,45].

## 3. Clinical Trials

Due to the role of *IDH1*/*IDH2* mutations in the pathogenesis of leukemia, as well as the promising results of preclinical studies indicating the potential ability of mIDH1/mIDH2 inhibitors to restore the normal differentiation of hematopoietic cells, it was decided to evaluate the effectiveness of the use of mIDH1/mIDH2 inhibitors in AML therapy in clinical trials. The results of the clinical trials performed are presented in Table 1.

The results of clinical trials assessing the effectiveness and safety profile of mIDH1/mIDH2 inhibitors in AML therapy are promising. In the case of patients with newly diagnosed or secondary mIDH2 AML, therapy with enasidenib was characterized by a higher complete remission (CR) rate compared to conventional care (23.4% and 3.7%, respectively) [56]. In turn, the use of ivosidenib in the treatment of patients with newly diagnosed mIDH1 AML allowed the achievement of CR in 30.3% of cases [47]. Thanks to the use of ivosidenib in a synergistic therapy with azacitidine, CR was achieved in 47% of cases [52]. Chen et al., in a meta-analysis analyzing the effectiveness of the use of mIDH1 inhibitors in the treatment of newly diagnosed AML, observed the approximately two times higher effectiveness of the combination therapy (CR—57%, overall response rate (ORR)—76%, 2-year overall survival (OS)—55%, 2-year event-free survival (EFS)—31%) compared to the use of mIDH inhibitors in a monotherapy (CR—23%, ORR—42%, 2-year OS—28%). However, it should be emphasized that the use of the combination therapy with mIDH inhibitors had comparable effectiveness to other intensive therapies used in the treatment of AML [58]. Based on the results of clinical trials, the Food and Drug Administration (FDA) has approved three mIDH inhibitors for the treatment of AML—see Table 2. The mechanism of action of mIDH inhibitors is based on the stabilization of the open conformation of mIDH, which causes the enzyme to lose its catalytic ability (which requires a closed conformation) [35].

It is necessary to conduct further clinical trials on larger groups of patients to assess the therapeutic benefits of using the synergistic therapy with mIDH inhibitors. Additionally, when the combination therapy is used, it is important to carefully assess the safety profile, as the use of several agents may intensify the adverse events to a level that is unacceptable to patients.

## 4. Challenges Associated with the Use of mIDH Inhibitors

### 4.1. Adverse Events

mIDH inhibitors have demonstrated relative safety in AML therapy [58,62]. The most common adverse events associated with their use that were recorded in clinical trials are presented in Table 1. It can be noted that the most frequently reported adverse events of Grade 3 or higher were hematological complications, such as thrombocytopenia, anemia, neutropenia or febrile neutropenia, while gastrointestinal (diarrhea, vomiting, increased alanine aminotransferase or aspartate aminotransferase levels), cardiological (prolongation of QT interval), pulmonological (pneumonia) and dermatological (rash) complications occurred less frequently. Another common adverse event characteristic of therapy with mIDH inhibitors is the potentially fatal differentiation syndrome (DS).

DS occurs not only when mIDH inhibitors are used. It was first described in the 1990s in patients with acute promyelocytic leukemia treated with all-trans retinoic acid (ATRA) [63,64]. The characteristic symptoms of this syndrome include dyspnea, fever, weight gain, hypotension, renal failure, pulmonary infiltrates and pleuropericardial effusions [63,64,65]. Based on these symptoms, Montesinos et al. presented diagnostic criteria according to which the presence of at least two of the aforementioned symptoms allows for the diagnosis of DS in patients treated with ATRA and anthracycline chemotherapy [66]. However, it is not known whether these criteria can be effectively applied in the case of DS caused by mIDH inhibitors [64]. The treatment of DS usually includes the administration of glucocorticosteroids, diuretics and hydroxyurea in the case of leukocytosis [47].

In the discussed clinical studies, DS of at least Grade 3 occurred with a frequency of 3.9–14.0% [46,55]. However, the researchers point out that this number may be significantly underestimated because this adverse event was not expected in the clinical trials; therefore, it was not initially assessed prospectively [64,67]. For this reason, Notsworthy et al. conducted an analysis to assess the actual incidence of DS in patients in two clinical studies [14]. The incidence of DS reported by the authors in these studies ranged from 11 to 14%. Notsworthy et al., based on the developed algorithm, identified 19% (34/179) of patients with DS treated with ivosidenib and 19% (41/214) of patients with DS treated with enasidenib [14]. In another analysis of the occurrence of DS in patients treated with enasidenib, its incidence was estimated at 10.4% (67/643). Additionally, it has been shown that a higher baseline blast count and lactate dehydrogenase may increase the risk of developing DS. The impact of DS on patients in achieving CR was also assessed. Interestingly, there was no relationship between DS and patients achieving CR, although such reports appeared in previous studies [11].

Therefore, the early detection of DS seems to be extremely important because its treatment methods are effective. However, not implementing the treatment early enough may result in the patient’s death. Further research is necessary to determine the safety profile of mIDH inhibitors in AML therapy, especially in the case of synergistic therapies, where the use of more therapeutic agents may lead to the intensification of the adverse events caused by individual drugs.

### 4.2. Resistance to mIDH Inhibitors

IDH inhibitors have demonstrated good efficacy in the treatment of AML in clinical trials, but their use remains limited by certain challenges. One of them is the development of resistance to these drugs in patients [68,69]. It has been shown that mIDH inhibitors effectively reduce the level of the oncometabolite 2-HG, but this does not translate into a clinical response in all patients, suggesting the occurrence of resistance to this treatment [70]. It may be either primary (resistance mechanisms reducing the effectiveness of mIDH inhibitor therapy were present before the start of treatment) or secondary (resistance mechanisms reducing the effectiveness of mIDH inhibitor therapy developed during the therapy). Primary resistance mechanisms include receptor tyrosine kinase (RTK) pathway mutations, mitogen-activated protein kinase (MAPK) pathway activation and epigenetic regulation, stemness and differentiation. In the context of the occurrence of primary resistance mechanisms, it seems important to select appropriate patients and conduct further research on prognostic factors that would allow the prediction of the effectiveness of therapy with mIDH inhibitors. Secondary resistance mechanisms include clonal evolution and selection, isoform switching, mitochondrial metabolism, RTK pathway mutations and secondary *IDH* mutations [68,69,71]. In the following section, we briefly discuss these resistance mechanisms and their clinical implications.

#### 4.2.1. Primary Mechanisms of Resistance to mIDH Inhibitors

It was shown that the presence of mutations in the RTK pathway in patients with R/R AML before the start of treatment was associated with a poorer response to this therapy. The authors of a clinical trial examining the use of ivosidenib in patients with R/R AML noted that baseline mutations in RTK pathway genes were more common in patients who did not achieve CR or achieved CR with partial hematologic recovery (CRh) [46]. Accordingly, in an extensive analysis by Choe et al. in the same group of patients, mutations in genes such as *NRAS*, *KRAS*, *PTPN11*, *KIT* and *FLT3* were identified, the co-occurrence of which with the *IDH1* mutation translated into a significantly lower probability of achieving CR after ivosidenib treatment [70]. Similarly, in another study in patients treated with enasidenib, mutations in the *NRAS* gene were more common in patients not responding to treatment with this mIDH inhibitor [72].

Another signaling pathway whose activation is also associated with resistance to mIDH inhibitors is the MAPK signaling pathway. Amatangelo et al. observed that patients with R/R AML who did not respond to enasidenib treatment were more likely to have mutations activating the MAPK pathway (including *NRAS*, *PTPN11*, *SRSF2*, *ASXL1*) [72]. Interestingly, in the case of co-occurring mutations within the *NRAS* gene, some patients achieved CR, which suggests that this mutation does not cause complete resistance to treatment with mIDH inhibitors [69,70,71].

Resistance to mIDH inhibitor therapy may also be determined by the population of cancer stem cells (CSCs), capable of self-renewal and long-term maintenance, which may result in the recurrence of the cancer after the completion of treatment [73]. The hypermethylation phenotype associated with the molecular factors forkhead box protein C1 (FOXC1), cluster of differentiation 99 (CD99) and DNA methyltransferase 3A (DNMT3A), as well as the presence of mutations in genes regulating the differentiation of the hematopoietic system (*RUNX1*, *CEBPA*, *GATA2*) in cancer cells, increases their stem cell-like phenotype (stemness). This results in a poorer response to treatment with mIDH inhibitors, resulting from the inability of dedifferentiated tumor cells to differentiate. Additionally, the *IDH* mutation itself increases the stemness phenotype in cancer cells through the dysregulation of the Wnt/β-catenin signaling pathway, dependent on the increased concentration of 2-HG. The increased activation of the Wnt/β-catenin pathway leads to the reduced differentiation of cancer cells and the maintenance of CSCs [68,69,71,73].

#### 4.2.2. Secondary Mechanisms of Resistance to mIDH Inhibitors

The appearance of subsequent somatic mutations in AML contributes to the dynamically changing heterogeneity of cancer cells. Due to the evolutionary pressure associated with the use of anticancer drugs, AML cells may acquire mutations that provide resistance to a given therapeutic agent, which will lead to the selection of resistant clones and ultimately the failure of the therapy. The potential risk of acquired resistance to mIDH inhibitor therapy is associated with the occurrence of mutations in the *U2AF1*, *RUNX1*, *BCORL1*, *GATA2*, *BCL11A*, *NFKB1*, *DDX1*, *MTUS1*, *DHX15* and *DEAF1* genes. An increase in the variant allele frequency of *CSF3R*, *FLT3* and *CBL* also predisposes patients to the development of resistance to treatment with mIDH inhibitors. In turn, deletion covering all or part of chromosome 7 in cancer cells carries the risk of developing acquired resistance to enasidenib. Additionally, during AML relapse after treatment with mIDH inhibitors, co-occurring mutations in the *KRAS*, *NRAS* and *TET2* genes are often reported. The accumulation of somatic mutations, resulting in the significant heterogeneity of AML, is one of the arguments supporting the need to use synergistic therapies [68,69,71].

Another mechanism of acquired resistance is isoform switching. Harding et al. reported three cases of patients with R/R AML and one patient with intrahepatic cholangiocarcinoma (ICC) in whom this phenomenon was observed [74]. In two patients with R/R AML with the *IDH1^R132C^* mutation, in whom a new increase in 2-HG in the blood was observed after initial remission, a new mutation—*IDH2^R140Q^*—was detected. In the case of the patient with ICC, the primary mutation was *IDH1^R132C^*, while, after recurrence, the *IDH2^R172V^* mutation was detected. In the last R/R AML patient with the *IDH2^R140Q^* mutation, treated with enasidenib, a previously absent *IDH1^R132C^* mutation was detected with the relapse of the disease. The authors hypothesize that the cause of this phenomenon may be the selective pressure to inhibit mIDH activity in one cellular compartment. This, in turn, may favor the survival of cancer cells with mIDH in another cellular compartment whose activity is not disturbed. Interestingly, in the case of the fourth patient, the use of combined mIDH1/2 blockade with AG-881 achieved a transient clinical response [74]. This indicates the need for further research on the simultaneous inhibition of both IDH isoforms using dual inhibitors.

IDH1/2 are important in many metabolic processes, including the Krebs cycle, OxPHOS and fatty acid synthesis [68,69]. Stuani et al. showed that mitochondrial oxidative metabolism was increased in AML cells with *IDH* mutation. This was related to CEBPα-induced fatty acid oxidation. The authors observed that mIDH inhibitors reduced 2-HG and CEBPα methylation, but did not reduce FAO or OxPHOS. This happened because the mIDH inhibitors abolished the inhibitory effect of 2-HG on TET2, and its activation contributed to maintaining high FAO and OxPHOS activity. Additionally, the authors examined the simultaneous use of mIDH inhibitors with OxPHOS inhibitors in vivo and proved that such a combination increased the effectiveness of the mIDH inhibitors [38].

The previously mentioned mutations in the RTK pathway may also be responsible for acquired resistance to mIDH inhibitors; however, the biological nature of the processes responsible for this phenomenon is not yet well studied [69]. In a study by Choe et al. on 26 patients who relapsed after achieving CR or CRh, nine (35%) had mutations in RTK pathway genes that were not identified at baseline [70]. The authors also tried to explain why mutations in the RTK pathway are associated with both primary and acquired resistance. Therefore, three hypotheses were put forward: (i) the activation of the RTK pathway is strong enough to reduce the dependence on 2-HG; (ii) the activation of the RTK pathway leads to a block in differentiation, which is additionally intensified by the use of mIDH inhibitors; and (iii) *IDH* mutations can cause the activation of the RTK pathway, and the use of an mIDH inhibitor would not reverse this activation in the case of co-occurring RTK pathway mutations [70].

Oltvai et al. reported a case of a patient treated with ivosidenib whose disease relapsed after an initial response to treatment. Even before the drug was administered, the *IDH1^R132C^* variant was identified in the patient. Due to the recurrence of the disease, the patient underwent genetic testing again and, in addition to the previously mentioned variant, another mutation in the *IDH1* gene—the p.S280F variant—was detected. All historical samples from the patient were then retrospectively examined, but the presence of the *IDH1^S280F^* variant before disease recurrence was not identified [75]. This is another example of acquired resistance, called a secondary *IDH* mutation. As a result of mutations in a site other than the primary mutation, the structure and interaction between mIDH and the inhibitor are disrupted, which translates into the ineffectiveness of the therapy [69].

## 5. Prospects for the Future

### 5.1. Strategies Aimed at Overcoming Isoform Switching

FDA-approved mIDH1/IDH2 inhibitors are specific to a given enzyme isoform, which promotes the development of resistance resulting from isoform switching. The aforementioned resistance mechanism can be overcome by the simultaneous blocking of both isoforms of the enzyme. Currently, the clinical effectiveness and safety profile of vorasidenib (AG-881), the first dual inhibitor of mIDH1 and mIDH2, in the treatment of glioma with *IDH1*/*IDH2* mutation are being investigated [24]. Other pan-IDH inhibitors of both mIDH1 and mIDH2 are the compounds LY3410738 and HMPL-306, the effectiveness of which is currently being tested in the therapy of IDH-mutated gliomas [76,77]. Using the potential of artificial intelligence (AI) to detect new compounds with physicochemical properties, enabling the simultaneous inhibition of IDH1 and IDH2, may lead to an increase in the number of pan-IDH inhibitors available on the market. Conducting further clinical trials with compounds identified by AI that bind to mutant enzymes in places with the most optimal space conformation may enable the selection of compounds with higher selectivity, effectiveness and safety than the currently available drugs [78].

### 5.2. Strategies Aimed at Overcoming Secondary IDH Mutations

Another mechanism of resistance to mIDH inhibitors is the formation of secondary *IDH* mutations. FDA-approved mIDH inhibitors inhibit mutant enzymes through allosteric binding at the interface of the IDH dimer, which leads to the enzyme adopting an open conformation, unable to carry out the reaction [79]. Secondary mutations occurring at the interface of the IDH dimer may cause resistance to drugs by limiting the stability of the enzyme–inhibitor connection [22,79]. For this reason, there is an ongoing search for new mIDH1/IDH2 inhibitors that would inhibit the mutant enzymes by binding to their active sites, which could reduce the resistance mechanism resulting from the formation of secondary *IDH* mutations [22]. Chaturvedi et al., in their work, reported the compound HMS-101, which inhibits mIDH1 by binding to it in the active site. The administration of HMS-101 to mouse models of AML increased the animals’ median survival by 20 days compared to the control group. Under the influence of HMS-101, cancer cells were characterized by the higher expression of transcription factors associated with differentiation, as well as the reduced presence of 2-HG and the resulting reduced histone methylation. Due to the lack of an FDA-approved mIDH inhibitor that binds to the active site of the enzyme, further studies of the safety profile and effectiveness of HMS-101 are necessary, as well as an intensive search for further compounds allowing the inhibition of mIDH by binding the active site of the enzyme [20,80].

**Figure 3:**
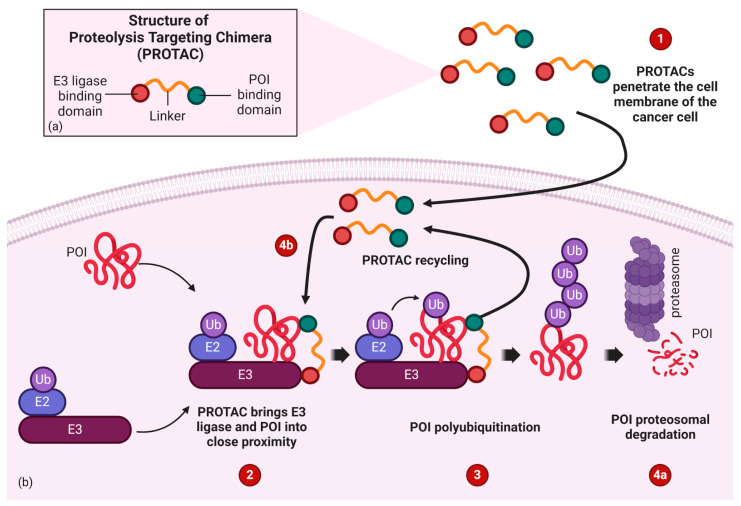
(**a**) PROTAC is a bifunctional molecule consisting of a ligand of the POI and a ligand of an E3 ubiquitin ligase (E3). The POI ligand and the E3 ligand are connected via a linker. (**b**) Mechanism of action: (1) PROTAC molecules penetrate the cell membrane; (2) upon POI binding to the ligand, PROTAC recruits E3 for (3) POI polyubiquitination, which ultimately leads to (4a) POI degradation, and (4b) the PROTAC molecule itself is recycled. PROTAC—proteolysis targeting chimera, E3—E3 ubiquitin ligase, E2—ubiquitin-conjugating enzyme, Ub—ubiquitin, POI—protein of interest. Image created with biorender.com (accessed on 11 July 2024).

Another potential strategy to overcome the effect of secondary IDH mutations is the use of targeted, post-translational protein degradation using proteolysis targeting chimera (PROTAC) technology, the structure of which, as well as the mechanism of action, is presented in Figure 3 [81,82].

Constructing a PROTAC whose protein of interest (POI) is IDH would lead to the PROTAC-mediated degradation of the mutant enzyme. The advantage of PROTAC is its ability to knock down POI by reducing its concentration in the cell, whereas classical inhibitors only inhibit POI’s activity itself. Therefore, PROTAC may be more effective than classical inhibitors, because restoring the function of the mutated IDH would require the re-synthesis of this protein in the cancer cell [82]. Additionally, thanks to the only transient, reversible binding of POI to PROTAC, this technology allows one to overcome the resistance mechanism consisting of the development of secondary mutations in POI. It is worth emphasizing, however, that mutations in the ubiquitin–proteasome system in cancer cells may lead to the development of resistance to PROTAC [81,82]. Other challenges associated with PROTAC technology are (i) the high cost of production, resulting from the numerous studies necessary to optimize the binding sites of PROTAC to POI, and (ii) the poor permeability of PROTAC into the cell due to the high molecular weight and large exposed polar surface of PROTAC. Therefore, further research on the modification of PROTAC is required so as to optimize the therapeutic effect in vivo [81,82]. The reduction of the costs of PROTAC construction can be partially achieved by using tools based on AI that enable the effective prediction of the optimal 3D structures of compounds, thus allowing the construction of PROTACs with the desired physicochemical properties [82]. Currently, however, research is necessary to construct a PROTAC whose POI would be IDH, as well as to determine the effectiveness of the aforementioned approach in the treatment of AML or glioma with an IDH mutation in vivo in preclinical studies.

However, it should be noted that the use of mIDH inhibitors in monotherapy usually leads, after some time, to the development of resistance and the subsequent recurrence of the cancer, despite the initial effectiveness of the therapy [83]. Improvements in the effectiveness of mIDH1/IDH2 inhibitor therapy may be achieved by using a synergistic approach. Currently, several drugs are being considered in order to improve the effectiveness of mIDH inhibitor therapy.

### 5.3. Strategies Aimed at Reducing DNA and Histone Methylation

Due to the inhibitory effect of 2-HG on the enzymes responsible for DNA and histone demethylation, therapies combining the use of mIDH inhibitors with hypomethylating agents are proposed. For example, the administration of ivosidenib with azacitidine, a hypomethylating drug, resulted in a clinical response in patients who, in addition to the *IDH1* mutation, also had mutations in genes considered to be unfavorable predictors of the response to ivosidenib (*NRAS*, *KRAS* and *PTPN11*) [36]. The synergistic administration of azacitidine with mIDH1 inhibitors additionally exerts an inhibitory effect on the MAPK/extracellular signal-regulated kinase (ERK) and RB/E2F pathways [6]. It has also been reported that cladribine, by reducing DNA methylation by limiting the available pool of the active methyl donor (S-adenosylmethionine), may act synergistically with mIDH2 inhibitors [84]. On the other hand, mIDH inhibitors may enhance the effects of other chemotherapeutic agents. Morell et al. showed that the use of enasidenib in combination with daunorubicin increased the effectiveness of daunorubicin by (i) inhibiting aldo-keto reductase 1C3 (AKR1C3), the expression of which in cancer cells promotes the development of insensitivity to anthracyclines; (ii) limiting the activity of ATP-binding cassette subfamily B member 1 (ABCB1), ATP-binding cassette subfamily G member 2 (ABCG2) and ATP-binding cassette subfamily C member 1 (ABCC1) transporters, which can pump chemotherapeutic agents out of the cancer cell. Enasidenib therapy may therefore provide therapeutic benefits in AML patients with *IDH2* mutations and AKR1C3 overexpression, especially when combined with anthracyclines [3]. A reduction in DNA hypermethylation can also be achieved by increasing TET’s activity. The TET-inhibitory effect of 2-HG can be overcome through the synergistic administration of mIDH inhibitors with TET inducers, which include vitamin C administered in high doses [85]. It is worth emphasizing, however, that the assessment of the effectiveness of the use of TET inducers, DNA/histone methyltransferase inhibitors or histone deacetylase inhibitors in the treatment of AML with IDH mutations requires further research [86].

### 5.4. Strategies Targeting Abnormal Gene Expression and Activation of Signaling Pathways

Abnormal DNA methylation causes the disruption of the 3D structure of chromatin, which may promote the expression of oncogenes. For example, the *IDH1^R132H^* mutation resulting in CpG hypermethylation in the CTCF region upstream of the PDGFRA gene locus leads to the abnormal interaction of the enhancer with the PDGFRA promoter and the upregulation of PDGFRA [12,28]. Therefore, cancer cells with the *IDH1^R132H^* mutation show increased sensitivity to the tyrosine kinase inhibitor dasatinib [12].

Due to the role that the RTK pathway plays in the development of resistance to mIDH inhibitors, the administration of therapeutic agents that inhibit the RTK pathway in combination with mIDH inhibitors is one of the potential therapeutic options [70]. However, taking into account the increased activation of the mTOR pathway in cells with the *IDH* mutation, one of the therapeutic options may be the use of the mTORC1 inhibitor rapamycin, which, by inhibiting GLS, could contribute to reducing the intracellular concentration of 2-HG [30].

An in vitro study on AML and primary human AML cell lines also demonstrated a beneficial, synergistic effect of combining enasidenib with ATRA. This approach (i) promoted cell differentiation due to the activation of the RAF-1/MEK/ERK pathway, (ii) induced autophagy and (iii) promoted histone demethylation. However, it should be noted that both ATRA and enasidenib as monotherapies may cause potentially fatal DS, and the incidence and severity of the DS symptoms could be increased if these drugs were administered synergistically. Moreover, potential clinical trials may be limited due to the safety profile of such a therapeutic approach [87].

### 5.5. Strategies Targeting Disturbed Cellular Metabolism

Taking into account disorders in cellular metabolism resulting from *IDH* mutations, several therapeutic strategies are being considered that could improve the effectiveness of mIDH inhibitor therapy. Thomas et al. showed that AML cells with the *IDH1* mutation had a limited ability to grow in lipid-poor conditions and also showed their dependence on the presence of acetyl coenzyme A carboxylase 1 (ACC1) (the knockdown of ACC1 resulted in the limited growth of cells with the *IDH1* mutation). It is worth emphasizing that the aforementioned relationships did not apply to cancer cells with *IDH2* mutations, which implies that mutations in IDH isoforms may modulate cellular metabolism in various ways, and understanding them is extremely important for personalized therapy. Therefore, the restriction of lipid intake and the pharmacological inhibition of ACC1 could offer a potential therapeutic strategy for *IDH1* mutations. It is worth noting, however, that limiting lipid intake may increase cancer-related cachexia and thus worsen the patient’s condition [37].

In addition to impaired lipid metabolism, cells with IDH mutations are characterized by increased oxidative metabolism. Therefore, the use of OxPHOS inhibitors—for example, inhibitors of electron transport chain complexes I, III and V—could provide a therapeutic benefit [38,68].

### 5.6. Strategies Targeting the Increased Susceptibility of mIDH Cancer Cells to Oxidative Stress

Due to the increased susceptibility of mIDH cancer cells to oxidative stress, the effect of mIDH inhibitors could be enhanced by combining them with a GLS inhibitor. Another way to inhibit glutamate metabolism is to use the GDH-3-epigallocatechin gallate (EGCG) inhibitor. The use of an Nrf2 inhibitor is also considered, the increased expression of which in cells with an *IDH* mutation is probably a compensatory mechanism allowing the maintenance of the intracellular GSH pool. The use of these three compounds could sensitize cancer cells with *IDH* mutations to oxidative stress thanks to limiting the pool of intracellular GSH [30].

### 5.7. Strategies Targeting the Limited Ability of mIDH Cancer Cells to Repair DNA

Due to the limited ability of cancer cells with the *IDH* mutation to repair double-stranded DNA breaks in the process of homologous recombination, they become dependent on enzymes from the poly(adenosine diphosphate-ribose) polymerase (PARP) family, the activity of which allows the cancer cell to maintain the integrity of its genome. The inhibition of PARP enzymes in mIDH cancer cells results in an increased number of single-strand DNA breaks and double-strand DNA breaks [30]. In vivo studies in a mouse model of AML have shown that the use of olaparib, a PARP inhibitor, demonstrates therapeutic effectiveness despite blast resistance to enasidenib [83]. The use of a synergistic therapy involving the administration of PARP inhibitors together with mIDH inhibitors could therefore provide therapeutic benefits [30,83]. Another drug that improves the effectiveness of mIDH inhibitor therapy is venetoclax, a selective inhibitor of the anti-apoptotic protein BCL-2. Cancer cells with *IDH* mutations depend on abnormally increased BCL-2 expression, which protects them from apoptosis. Therefore, the combination of venetoclax with mIDH inhibitors results in the increased sensitivity of leukemic blasts to apoptosis via the intrinsic pathway [30,42]. An increase in the effectiveness of venetoclax treatment can be additionally achieved by using verapamil, an ABCB1/MDR1/P-GP efflux pump inhibitor [12].

### 5.8. Currenly Recruiting Clinical Trials

At the time of writing, there are nine currently recruiting clinical trials testing the efficacy and safety of mIDH inhibitors both in monotherapy and with other agents. For more information, see Table 3.

## 6. Conclusions

mIDH1/IDH2 inhibitors are a promising therapeutic option for R/R AML patients with an *IDH1*/*IDH2* mutation. This is particularly important in the case of geriatric patients, in whom *IDH* mutations are more common and who (due to comorbidities) cannot be included in the treatment of choice based on intensive chemotherapy. Therapeutic benefits from the use of these drugs may also be obtained by patients with acute lymphoblastic leukemia with an *IDH1/2* mutation or patients with myeloproliferative neoplasms (MPN), in which the presence of an *IDH1/2* mutation may be associated with an increased risk of MPN transforming into AML due to the impaired differentiation of myeloid progenitor cells [79,83]. One of the challenges limiting the effectiveness of mIDH inhibitor therapy in clinical practice is the heterogeneity of AML. To achieve a better clinical response when using mIDH inhibitors, it is necessary to implement a more individualized therapy—based on the results of molecular profiling determining not only the occurrence of *IDH* mutations in leukemic blasts but also the presence of primary resistance mechanisms. Improvements in the response to mIDH inhibitor therapy may also be achieved by using a synergistic approach, which could reduce the emergence of secondary resistance mechanisms. Further studies are necessary to evaluate the therapeutic effectiveness of the combination of mIDH inhibitors with hypomethylating drugs and the inhibitors of tyrosine kinase, mTORC1, OxPHOS, GLS, Nrf2, PARP and BCL-2, in AML therapy. It is also important to have a better understanding of the molecular mechanisms by which *IDH* mutations promote the pathogenesis of AML, which will enable the search for new molecular targets that could improve the effectiveness of *mIDH* inhibitor therapy.

## Figures and Tables

**Table 1:** Results of clinical trials conducted so far assessing the use of mIDH inhibitors in the treatment of AML.

Illness	Study Phase	Treatment Protocol (Drug and Dose)	Number of Patients and Their Median Age (Range)	Outcome	Most Common Adverse Events of Grade 3 or Higher	Reference
R/R AML	Phase 1	Ivosidenib Starting dose: 500 mg/day	179; 67.0 years (18–87)	CR—21.8% (39/179); CRi or CRp—11.7% (21/179); MLFS or bone marrow CR—5.6% (10/179); SD—38.5% (69/179); PD—8.4% (15/179)	Prolongation of the QT interval on ECG—7.8%; IDH differentiation syndrome—3.9%; Anemia—2.2%; Thrombocytopenia—1.7%; Leukocytosis—1.7%; Platelet count decreased—1.7%	[46]
Patients with newly diagnosed *IDH1* AML	Phase 1	Ivosidenib: 500 mg/day	34; 76.5 years (64–87)	CR—30.3% (10/33); CRi/CRp—18.2% (6/33); PR—3% (1/33); MLFS—3% (1/33); SD—30.3% (10/33); PD—9.1% (3/33)	IDH differentiation syndrome—9%; Prolongation of the QT interval on ECG—6%; Febrile neutropenia—6%; Diarrhea—6%	[47]
R/R AML	Phase 1/2	Enasidenib: 100 mg/day	214; 68.0 years (19–100)	CR—19.6% (42/214); CRi/CRp—9.3% (20/214); PR—4.2% (9/214); MLFS—5.6% (12/214); SD—45.8% (98/214); PD—8.9% (19/214)	Hyperbilirubinemia—10.4%; Thrombocytopenia—6.7%; IDH differentiation syndrome—6.4%; Anemia—5.5%	[48]
Patients with newly diagnosed AML	Phase 1/2	Enasidenib: 50–650 mg/day	39; 77.0 years (58–87)	CR—18% (7/39); CRi/CRp—3% (1/39); PR—5% (2/39); MLFS—5% (2/39); SD—49% (19/39); PD—3% (1/39)	Indirect hyperbilirubinemia—13%; Anemia—13%; IDH differentiation syndrome—10%; Thrombocytopenia—8%; Tumor lysis syndrome—8%	[49]
Patients with *IDH1* AML	Phase 1/2	Olutasidenib: 150 mg, twice daily	147; 71.0 years (32–87)	CR/CRh—35% (51/147); CRi—10% (15/147); PR—2% (3/147); MLFS—1% (2/147); SD—29% (42/147); PD—7% (10/147)	Febrile neutropenia—20%; Anemia—20%; Thrombocytopenia—16%; Neutropenia—13%; Leukocytosis—9%; IDH differentiation syndrome—8%	[50]
Patients with newly diagnosed m*IDH1* AML	Phase 1b	Ivosidenib: 500 mg/day; Azacitidine: 75 mg/m^2^/day for 7 d/cycle	23; 76.0 years (61–88)	CR—60.9% (14/23); CRi/CRp—8.7% (2/23); MLFS—8.7% (2/23); SD—17.4% (4/23)	Neutropenia—22.0%; Anemia—13.0%; Thrombocytopenia—13.0%; Prolongation of the QT interval on ECG—13.0%	[51]
Patients with newly diagnosed *IDH1* AML	Phase 3	Ivosidenib: 500 mg/day; Azacitidine: 75 mg/m^2^/day for 7 d/cycle	72; 76.0 years (58–84)	CR—47% (34/72); CRi/CRp—7% (5/72); PR—6% (4/72); MLFS—3% (2/72); SD—10% (7/72); PD—3% (2/72)	Febrile neutropenia—28%; Neutropenia—27%; Anemia—25%; Thrombocytopenia—24%; Pneumonia—23%	[52]
Patients with newly diagnosed m*IDH1/2* AML	Phase 1	Ivosidenib: 500 mg/d and chemotherapy (cytarabine: 200 mg/m^2^/day for 7 days with either daunorubicin 60 mg/m^2^/day for 3 days or idarubicin 12 mg/m^2^.d for 3 days)	60; 62.5 years (24–76)	CR—68% (41/60); CRi/CRp—8% (5/60); PR—3% (2/60); MLFS—7% (4/60); Treatment failure—13% (8/60)	During induction period: Hypophosphatemia—16.7%; Hypokalemia—11.7%; Electrocardiogram QT prolonged—10.0% During consolidation period: rash, vomiting, hypokalemia, pyrexia, electrocardiogram QT prolonged, ALT increased, headache, hypoalbuminemia, blood bilirubin increased, hypocalcemia (all occurring once—2.9%)	[53]
		Enasidenib: 100 mg/day and chemotherapy (same as above)	93; 63.0 years (27–77)	CR—55% (50/91); CRi/CRp—19% (17/91); PR—2% (2/91); MLFS—11% (10/91); Treatment failure—13% (12/91)	During induction period: Blood bilirubin increased—16.1%; Rash—14%; Hypophosphatemia—12.9% During consolidation period: Blood bilirubin increased—10.9%; Stomatitis—8.7%; Hypokalemia—8.7%; Hypophosphatemia—8.7%	
	Phase 2	Enasitenib: 100 mg/day; Azacitidine: 75 mg/m^2^/day for 7 d/cycle	68; 75.0 years (70–79)	CR—54% (37/68); CRi/CRp—9% (6/68); PR—6% (4/68); MLFS—4% (3/68); SD—19% (13/68); PD—1% (1/68)	Neutropenia—37%; Thrombocytopenia—37%; Anemia—19%; Febrile neutropenia—16%; IDH differentiation syndrome—10%	[54]
		Azacitidine: 75 mg/m^2^/day for 7 d/cycle	33; 75.0 years (71–78)	CR—12% (4/33); CRi/CRp—18% (6/33); PR—6% (2/33); SD—48% (16/33); PD—3% (1/33)	Neutropenia—25%; Anemia—22%; Thrombocytopenia—19%; Febrile neutropenia—16%; Pneumonia—6%; Lung infection—6%	
Patients with newly diagnosed AML or with R/R AML	Phase 2	Enasidenib: 100 mg/day; Azacitidine: 75 mg/m^2^/day for 7 d/cycle	Newly diagnosed AML—7; 77.0 years (66–81)	CR—72% (5/7); CRi—28% (2/7)	Blood bilirubin increased—29%; Febrile neutropenia—14%; IDH differentiation syndrome—14%	[55]
			R/R AML—19; 64.0 years (24–88)	CR—26% (5/19); CRi—32% (6/19); No response—37% (7/19)	Blood bilirubin increased—37%; Febrile neutropenia—26%; IDH differentiation syndrome—5%; Increased ALT level—5%; Increased AST level—5%	
Patients with newly diagnosed or secondary *IDH2* AML	Phase 3	Enasidenib: 100 mg/day	158; 72.0 years (60–85)	CR—23.4% (37/158); CRi/CRp—6.3% (10/158); PR—4.4% (7/158); MLFS—6.3% (10/158); SD—40.5% (64/158); PD—8.2% (13/158)	Thrombocytopenia—10.2%; Blood bilirubin increased—8.3%; Neutropenia—5.7%; IDH differentiation syndrome—5.1%; Anemia—4.5%	[56]
		CCR (azacitidine: 75 mg/m^2^/d for 7 d/cycle; or IDAC: 0.5–1.5 g/m^2^/day for 3–6 days per cycle; or LDAC 20 mg twice/day for 10 days per cycle; or BSC only)	161; 71.0 years (60–86)	CR—3.7% (6/161); CRi/CRp—2.5% (4/161); PR—0.0% (0/161); MLFS—3.7% (6/161); SD—33.5% (54/161); PD—18.0% (29/161)	Febrile neutropenia—12.1%; Neutropenia—10.6%; Thrombocytopenia—8.5%; Anemia—5%; Pneumonia—4.3%	
Patients with IDH1^R132^-mutant AML and MDS	Phase 1	IDH305 75–750 mg twice daily	41; 71.0 years (29–85)	CR—18.9% (7/37); CRi—8.1% (3/37); NR—37.8% (14/37); TF—24.3% (9/37)	Febrile neutropenia—29.3%; Lung infection—29.3%; Fatigue—9.8%	[57]

mIDH—mutant isocitrate dehydrogenase, R/R AML—relapsed or refractory acute myeloid leukemia, CR—complete remission, CRh—complete remission with partial hematologic recovery, CRi—complete remission with incomplete hematologic recovery, CRp—complete remission with incomplete platelet recovery, MLFS—morphologic leukemia-free state, SD—stable disease, PD—progressive disease, PR—partial response, ECG—electrocardiogram, IDH—isocitrate dehydrogenase, ALT—alanine aminotransferase, AST—aspartate aminotransferase, CCR—conventional care regimen, IDAC—intermediate-dose cytarabine, LDAC—low-dose cytarabine, BSC—best supportive care, MDS—myelodysplastic syndrome, NR—no response, TF—treatment failure.

**Table 2:** mIDH inhibitors for the treatment of AML approved by the FDA.

Drug	Target Mutation	Indications	FDA Approval Date	Reference
Enasidenib (AG-221)	*IDH2*	Patients with R/R AML with *IDH2* mutation.	August 2017	[59]
Ivosidenib (AG-120)	*IDH1*	Patients at least 75 years old or with comorbidities that preclude the use of intensive chemotherapy, with ND AML with a susceptible *IDH1* mutation.	May 2019	[60]
Olutasidenib (FT-2102)	*IDH1*	Patients with R/R AML with a susceptible *IDH1* mutation.	December 2022	[61]

mIDH—mutated isocitrate dehydrogenase, AML—acute myeloid leukemia, FDA—Food and Drug Administration, ND—newly diagnosed, R/R AML—relapsed/refractory AML.

**Table 3:** Currently recruiting clinical trials evaluating mIDH inhibitors in AML.

Drug	Study Phase	Patient Population	Estimated Number of Patients	Clinical Trials.gov Identifier	Reference
Enasidenib + Cobimetinib (MEK inhibitor)	Phase 1b	Patients with R/R AML with co-occurring *IDH2* and *RAS* mutations	15	NCT05441514	[88]
Ivosidenib or Enasidenib + Azacitidine and Venetoclax	Phase 2	Patients with newly diagnosed *IDH1*/*IDH2* AML	125	NCT05401097	[89]
HMPL-306 (dual mIDH1/2 inhibitor)	Phase 3	Patients with *IDH*-mutant R/R AML	316	NCT06387069	[90]
HMPL-306	Phase 1	Patients with *IDH*-mutant advanced relapsed, refractory or resistant hematological malignancies	75	NCT04764474	[91]
Ivosidenib	Phase 1	Patients with *IDH1*-mutant advanced hematologic malignancies	291	NCT02074839	[92]
Ivosidenib or Enasidenib with induction and conslodiation therapy followed by maintenance therapy	Phase 3	Patients with *IDH1*/*IDH2*-mutant newly diagnosed AML or MDS	968	NCT03839771	[93]
Ivosidenib + CPX-351 (liposome-encapsulated daunorubicin-cytarabine)	Phase 2	Patients with *IDH1*-mutant AML or high-risk MDS	30	NCT04493164	[94]
Decitabine/Cedazuridine + Venotoclax in combination with Ivosidenib/Enasidenib	Phase 1b/2	Patients with R/R AML	84	NCT04774393	[95]
Ivosidenib and combination chemotherapy	Phase 1	Patients with *IDH1*-mutant R/R AML	25	NCT04250051	[96]

mIDH—mutant isocitrate dehydrogenase, R/R AML—relapsed or refractory acute myeloid leukemia, IDH1/2—isocitrate dehydrogenase 1/2, MEK—mitogen-activated protein extracellular kinase, RAS—oncogenic rat sarcoma virus, MDS—myelodysplastic syndrome.

## Data Availability

Data are contained within the article.
